# Neuronanomedicine: An Up-to-Date Overview

**DOI:** 10.3390/pharmaceutics11030101

**Published:** 2019-02-26

**Authors:** Daniel Mihai Teleanu, Cristina Chircov, Alexandru Mihai Grumezescu, Raluca Ioana Teleanu

**Affiliations:** 1 Emergency University Hospital, “Carol Davila” University of Medicine and Pharmacy, 050474 Bucharest, Romania; daniel.teleanu@umfcd.ro; 2 Faculty of Engineering in Foreign Languages, University Politehnica of Bucharest, 060042 Bucharest, Romania; cristina.chircov@yahoo.com; 3 Department of Science and Engineering of Oxide Materials and Nanomaterials, Faculty of Applied Chemistry and Materials Science, Politehnica University of Bucharest, 060042 Bucharest, Romania; 4 ICUB—Research Institute of University of Bucharest, University of Bucharest, 36-46 M. Kogalniceanu Blvd., 050107 Bucharest, Romania; 5 “Dr. Victor Gomoiu” Clinical Children’s Hospital, “Carol Davila” University of Medicine and Pharmacy, 050474 Bucharest, Romania; raluca.teleanu@umfcd.ro

**Keywords:** neuronanomedicine, nanotechnology, neurological sciences, central nervous system disorders, nano-vehicles, organic nanocarriers, inorganic nanocarriers, delivery of imaging and therapeutic agents

## Abstract

The field of neuronanomedicine has recently emerged as the bridge between neurological sciences and nanotechnology. The possibilities of this novel perspective are promising for the diagnosis and treatment strategies of severe central nervous system disorders. Therefore, the development of nano-vehicles capable of permeating the blood–brain barrier (BBB) and reaching the brain parenchyma may lead to breakthrough therapies that could improve life expectancy and quality of the patients diagnosed with brain disorders. The aim of this review is to summarize the recently developed organic, inorganic, and biological nanocarriers that could be used for the delivery of imaging and therapeutic agents to the brain, as well as the latest studies on the use of nanomaterials in brain cancer, neurodegenerative diseases, and stroke. Additionally, the main challenges and limitations associated with the use of these nanocarriers are briefly presented.

## 1. Introduction

Neuroscience is a multidisciplinary field that studies the macro- and microscale neuroanatomy, the functional organization of specific brain areas, and the electrophysiology of neurons and synapses. Moreover, neuroscience represents the means to understand the underlying mechanisms involving the structure and function of individual channels and receptor proteins, the development and repair signaling, and the assembly of proteins into molecular machines that regulate neuronal functions [1]. A complete knowledge of brain function is fundamental for developing novel and efficient strategies that will allow for the long-term and minimally invasive diagnosis and treatment of neurological diseases [1,2].

Neurological disorders comprise a wide variety of sporadic and hereditary [3] pathological conditions, including brain cancer, neurodegenerative diseases, multiple sclerosis, and stroke, which can manifest mild to severe symptoms [4,5]. Due to the increase in elderly populations, the prevalence of these diseases is becoming a great concern [5]. Neurological pathologies are characterized by processes involving protein aggregation which subsequently lead to neurodegeneration or dysregulation of immune mechanisms, or by the progressive loss of neuronal structure and function, associated with abnormalities in brain development and function and neuronal death [6,7]. However, diagnosis, management, and monitoring strategies for neurological disorders are currently unsuccessful mostly due to the complexity of the nervous system [8,9]. Additionally, their diagnosis and treatment involve high precision, dedication, and experience [9].

Furthermore, the presence of the blood–brain barrier (BBB) and the blood–cerebrospinal fluid barrier (BCSFB) represents the main cause for limitations in the management of neurological diseases [7]. The BBB comprises the neurovascular unit which includes specialized endothelial cells, pericytes, astrocytes, neurons, and the extracellular matrix [10]. The BBB represents the dynamic interface between the brain and the circulating blood, acting as a gateway to protect the brain from toxins and cells and to maintain its proper microenvironment [11,12] through the tight junctions, an intricate system of proteins between the endothelial cells [10]. By contrast, the BCSFB consists in epithelial cells only, which are responsible for the physical and chemical properties. Similarly, the tight junctions between the epithelial cells prevent the paracellular diffusion of molecules into the cerebrospinal fluid. If the molecules penetrate the barrier, they may enter into the interstitial fluid of the brain [13]. Since the delivery of contrast and therapeutics is restricted by the two barriers, the need to design novel approaches that can effectively target and reach the central nervous system is fundamental for the diagnosis and treatment of brain disorders [7].

Advances in nanotechnology have allowed for a better understanding of the pathological conditions of the nervous system and the development of formulations that could enhance the therapy of neurological diseases [14]. Comprising knowledge from multiple disciplines, including chemistry, physics, engineering, and biology [15,16], nanotechnology is defined as the field which aims to control matter at atomic and molecular levels [17,18,19]. The nanotechnology processes, also termed as nanofacture or ultraprecision engineering [20], have allowed for the development of non-invasive approaches for the delivery of therapeutic and imaging agents across the brain barriers [21,22]. Therefore, the combination of nanotechnology, specifically nanomedicine, and neuroscience has led to the birth of a novel field, neuronanomedicine, through which nanomaterials, nanoformulations, and nanofacturing processes are effectively employed in neurology for understanding physiological and pathological mechanisms and for diagnosing and treating the disorders of the central nervous system.

**Figure 1:**
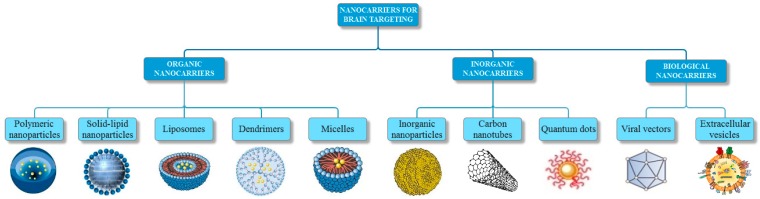
A summary of the various types of nanocarriers for brain targeting.

The scientific interest in designing nanotechnology-based approaches, including nanoparticles, liposomes, dendrimers, micelles, carbon nanotubes, quantum dots, viral vectors, and extracellular vesicles, which have the potential to deliver the appropriate amount of the therapeutic and imaging agent to the brain, has grown rapidly [21,23]. Hence, the subject of this paper is to review the nanocarriers presently used to diagnose and treat the most prevalent neurological disorders (Figure 1).

## 2. Nanocarriers for Brain Targeting

**Figure 2:**
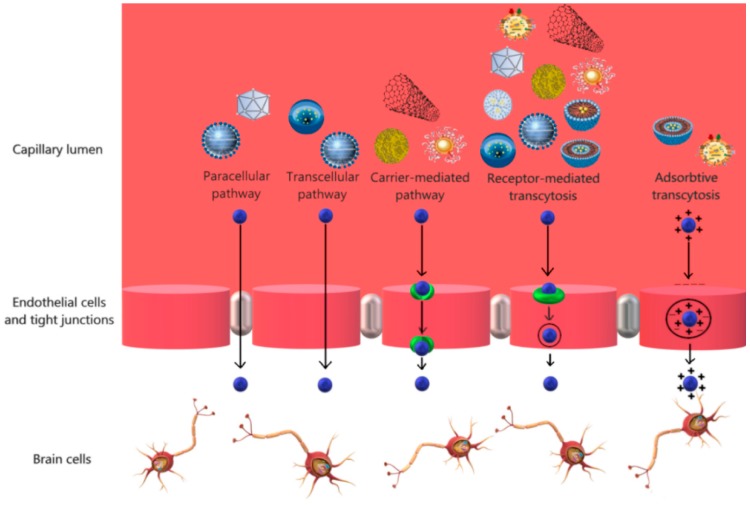
The main pathways of crossing the BBB for each type of the described nanocarriers.

Delivering drugs to the brain represents a challenge because conventional neuropharmaceuticals do not possess the appropriate physicochemical characteristics regarding molecular size, lipid solubility, and surface charge [24]. Hence, owing to the capacity to modulate their interactions with endothelial cells in the brain through surface functionalization, various nanocarriers have been employed [25]. Therefore, the encapsulation of therapeutic and imaging agents into specific nanocarriers might overcome the challenges associated with the conventional delivery methods across the BBB [26]. Additionally, after intravenous administration, nanocarriers are capable of crossing the tissues in the organism and reach the central nervous system [27]. A summary of the advantages and disadvantages associated with each nanocarrier type, as well as the surface functionalization strategies is presented in Table 1. Furthermore, the specific pathways to cross the BBB for each nanocarrier is presented in Figure 2.

### 2.1. Organic Nanocarriers

#### 2.1.1. Polymeric Nanoparticles

Polymeric nanoparticles used as nanocarriers involve matrix architectures, most commonly in the form of nanocapsules and nanospheres [28,29]. The most widely used polymers for manufacturing these nanocarriers are biocompatible and biodegradable and of synthetic origin, such as polylactic acid, polyglycolic acid, polylactide-co-polyglycolic acid, poly(ε-caprolactone), and polymethyl methacrylate, and of natural origin, such as chitosan, alginate, gelatin, and albumin [28,30]. The pharmacokinetics of the encapsulated agents is mainly influenced by the structure of the polymer and the entrapping method [31].

The mechanisms for brain uptake and drug release of polymeric nanoparticles have been intensively studied in order to design nanocarriers that can efficiently deliver therapeutics to the central nervous system through systemic and local administration. Hence, the main strategies involve endocytosis or transcytosis through the endothelial cells, accumulation in the brain capillaries resulting in the transfer to the brain parenchyma owing to the high concentration gradient, membrane fluidization through lipid solubilization due to the surfactant effect, tight junctions opening [32], and restricted efflux phenomenon by coating polymers with polysorbates [31]. Furthermore, to improve the transcytosis across the BBB, the surface of the polymeric nanoparticles can be functionalized by the conjugation of targeting peptides or cell-penetrating ligands [32].

#### 2.1.2. Solid-Lipid Nanoparticles

Solid-lipid nanoparticles are the new generation of colloidal nanocarriers consisting of surfactant-stabilized triglycerides, monoglycerides, hard fats, complex glyceride mixtures, or waxes, that are solid at both room and body temperatures [28,33,34]. Their structure usually involves a hydrophobic solid matrix core in which phospholipids are embedded through the hydrophobic tail regions. Therefore, the entrapment efficiency for hydrophobic drugs in the core is higher than conventional nanocarriers [33]. Commonly used solid lipids for the formulation of these nanocarriers are stearic acid, cetyl alcohol, cholesterol butyrate, carnauba wax, beeswax, and emulsifying wax [34,35].

With a size similar to other nanocarriers, between 50 and 1000 nm [35], solid-lipid nanoparticles combine the advantages of liposomes and polymeric nanoparticles, while outcoming the associated individual disadvantages [33]. Hence, solid-lipid nanoparticles are characterized by biocompatibility, high physical stability, bioavailability, drug protection, strict control of release, ease of preparation, good tolerance, and biodegradability without generating toxic by-products [36,37].

As they have the capacity to target the central nervous system and naturally cross the BBB due to their highly lipophilic nature, solid-lipid nanoparticles have been extensively used as nano-vehicles for the delivery of chemotherapeutic drugs into the central nervous system [37,38]. The main mechanisms involved in the brain uptake of solid-lipid nanoparticles are the paracellular pathway through the opening of the tight junctions in the brain microvasculature, passive diffusion, active transport, and endocytosis [39]. Moreover, since apolipoprotein E receptors are predominantly expressed in the brain, the functionalization of solid-lipid nanoparticles with this protein has become an important strategy in enhancing the brain targeted drug delivery [38,40,41].

#### 2.1.3. Liposomes

Liposomes are artificial and spherical vesicles, consisting of single or multiple amphiphilic lipid bilayers which surround an aqueous solution core [42,43,44,45]. As they can entrap both hydrophilic and hydrophobic compounds in the aqueous core and in the phospholipid bilayers, respectively [42,43], they have been extensively used as drug delivery systems to improve the safety and efficiency of therapeutics targeting. Hence, liposomes have been formulated as nanocarriers to efficiently deliver therapeutic molecules, including drugs, vaccines, enzymes, proteins, and nucleic acids [42], and imaging agents for diagnostics [46].

Furthermore, liposomes have demonstrated their potential in neurological applications as they can cross the BBB through passive or active targeting and deliver the appropriate quantity of therapeutic and diagnosis agents to the brain [47]. The main paths for liposomes to reach the brain parenchyma include the adsorption-mediated transcytosis, the receptor-mediated endocytosis, and the disruption of the BBB through external forces [48].

#### 2.1.4. Dendrimers

Dendrimers are a class of nanoscaled artificial, highly branched, globular macromolecules [49,50]. Their tree-like topological structure includes an initiator core, branched repeat units from the core, and functional terminal groups on the external layer of the repeat units [49]. The most common molecules for dendrimer formulations are polyamidoamine, polypropylenimine, and polyaryl ether [50]. As they are able to encapsulate both hydrophilic and hydrophobic molecules, these unrivalled polymer-based nanostructures [51] have been extensively used as nanocarriers to transport various therapeutic and imaging agents [50,52].

Dendrimers possess the capacity to overcome the BBB, and therefore, they have been widely applied in the therapy of central nervous system disorders [53]. Furthermore, they have the ability to cross various cell membranes or biological barriers through the endocytosis-mediated cellular internalization. Specifically, the cellular uptake is mediated by the reversible modulation of the tight junction proteins, such as occludin and actin. Moreover, specific ligands can be conjugated to the surface of the dendrimers for an enhanced brain targeting and facilitated transport across the BBB [54].

#### 2.1.5. Micelles

Micelles are amphiphilic nanocarriers with a particle size within the range of 5–50 nm that spontaneously form under certain conditions of concentration and temperature of the aqueous solution [55]. The mechanisms for generating micelles mainly involve the self-assembly of amphiphilic molecules. Their architecture is characterized by a core formed by the hydrophobic/non-polar regions of the molecules, known as the tail, and the outside surface comprising the hydrophilic/polar regions of the molecules, known as the head. Therefore, micelles have gained great scientific interest due to their ability to deliver poorly water-soluble and lipophilic compounds and their potential to improve drug bioavailability by providing chemical and physical stability and a sustained and controlled release [35].

Micelles penetrate the BBB mainly through the mechanisms of endocytosis and/or transcytosis. Furthermore, the penetration capacity can be enhanced by conjugating specific ligands and antibodies or by applying external thermal or mechanical forces to disrupt the BBB [56].

### 2.2. Inorganic Nanocarriers

#### 2.2.1. Inorganic Nanoparticles

The significant amount of work in the area of inorganic nanoparticles synthesis and surface modification has contributed immensely to their applicability in the medical field [57]. Moreover, their unique intrinsic optical, electrical, and magnetic properties have paved the way for novel biomedical applications, such as targeted drug delivery, cancer therapy, bioimaging, and biosensing [57,58].

Inorganic nanoparticles, specifically metal, semiconductor, and metal oxide nanoparticles, have attracted great scientific interest owing to the possibility of tuning their size, shape, composition, structure, and porosity and to decorate their surface to facilitate the conjugation of ligands and polymers, thus enhancing their biological performances [58,59]. As they lack the property of biodegradability, silver, iron oxide, and titanium oxide have been mostly applied for tissue bioimaging in disease diagnosis. However, several inorganic nanoparticles, such as gold and silica nanoparticles, have been used as nanocarriers across the BBB [60]. Moreover, as superparamagnetic iron oxide nanoparticles (SPIONs) are relatively large in size with a mean particle diameter higher than 50 nm, and exhibit unfavorable pharmacokinetic behavior that leads to liver and spleen accumulation due to the opsonization and scavenging by the mononuclear phagocyte system, ultra-small SPIONs (USPIONs) have been developed [61] for drug delivery applications.

Furthermore, to increase brain uptake of these nanoparticles, the application of external stimuli, including near-infrared radiation and magnetic field, could enhance the on-demand drug release across the BBB and improve tissue imaging. Moreover, inorganic nanoparticles are characterized by a prolonged enhanced permeability and retention effect which makes them a great candidate for brain cancer therapy [59].

#### 2.2.2. Carbon Nanotubes

Carbon nanotubes are the most commonly used among the class of carbon-based nanomaterials, comprising graphite sheets rolled into tubes with diameters within the nanoscale. Depending on their architecture, carbon nanotubes can be single-walled or multi-walled, with open ends or closed with fullerene caps [62]. Carbon nanotubes have gained great scientific attention in various fields owing to their unique structure, exceptional electrical, mechanical, optical, and thermal properties, and high surface area [63,64]. Their main nanomedical applications involve drug, hormone, and enzyme delivery, gene therapy, tissue engineering, and biosensing [64,65]. Owing to the possibility of functionalization using specific chemical compounds to modify their physical and biological properties, carbon nanotubes have been applied as nanocarrier systems [66]. As they cannot cross the BBB through passive diffusion, the conjugation of compounds that could facilitate the active transport to the brain is essential for the emerging applications in neuronanomedicine.

#### 2.2.3. Quantum Dots

Quantum dots are zero-dimensional nanomaterials which have attracted considerable scientific interest owing to their exceptional optical and electrical properties [67]. Their application in the fields of medicine and biology has emerged as nanoscaled systems for drug delivery, targeted cancer therapy, bioimaging, and transplanted cell labeling and tracking [68]. Similar to carbon nanotubes, quantum dots require subsequent surface functionalizations through which brain targeting and BBB crossing could be possible. Thus, the mechanisms for reaching the brain parenchyma mostly involve the carrier-mediated transport.

### 2.3. Biological Vectors

#### 2.3.1. Viral Vectors

The use of viral vectors is based on the ability of viruses to enter and insert genetic material into the host’s cells. The application of viral vectors in the central nervous system is mainly represented by gene therapy, which usually involves the delivery of a normal copy of a defective gene and the reduction of the deleterious functions [69]. The most intensively studied and commonly applied in clinical trials for gene therapy and cancer oncolytic therapy are retrovirus vectors, lentivirus vectors, adenovirus vectors, herpes simplex virus type 1, and adeno-associated virus vectors, which possess transgene capacity and expression properties [70].

The main strategies for the transportation of viral vectors across the BBB are the receptor-mediated pathway across the endothelial cells by transcytosis and the transient disruption of the BBB, which allows for the paracellular transport into the brain parenchyma. One method of disruption involves the intravenously administration of a highly concentrated mannitol solution which will result in the osmotic shrinkage of the cells [71].

Studies reported the use of herpes simplex viral vectors to combat stroke by repairing or replacing genes that lead to neuronal damage. Similar results were obtained by using the adenovirus-mediated vectors. Although there are promising solutions and results, gene therapy is still in its infancy due to ethical issues and high risks of therapy failure [72].

#### 2.3.2. Extracellular Vesicles

Extracellular vesicles represent a heterogenous class of cell-derived membrane structures, originating from the endosomal system, termed as exosomes, or shedding from the plasma membrane, termed as microvesicles. Extracellular vesicles can be ubiquitously found in biological fluids and have a role in various physiological and pathological processes [73]. Recently, they have attracted great attention as an additional mechanism for intercellular communication throughout the body, allowing for protein, lipid, and genetic material exchange [73,74].

In the central nervous system, extracellular vesicles are involved in the maintenance of normal neuronal functions and the development of neurodegenerative disorders. The pathways of BBB crossing by extracellular vesicles are mainly through adsorptive-mediated transcytosis or receptor-mediated transcytosis. However, the underlying and precise mechanisms of crossing in physiological and pathological conditions are not completely understood [74].

The applications of extracellular vesicles as nanocarriers for brain disorders include the use of autologous exosomes containing glyceraldehyde-3-phosphate dehydrogenase that can deliver small-interfering RNA to neurons, microglia, and oligodendrocytes. Moreover, they were also used for the delivery of the APP cleaving enzyme for the downregulation of the BACE1 protein. The intranasal administration of curcumin-containing exosomes for the inhibition of brain inflammation and autoimmune responses has been reported. Exosomes can also be used to deliver Stat3 inhibitor JSI-124 to inhibit tumor growth in a glioblastoma model [75].

Considering the abovementioned characteristics, solid-lipid nanoparticles might be preferred for the treatment applications using hydrophobic drugs as they are highly lipophilic, biodegradable, there are no neurotoxic effects reported in the literature, and they can cross the BBB through the paracellular pathway. Nevertheless, if the nature of the therapeutic agent is hydrophilic, polymeric nanoparticles can be applied, but their reported neurotoxicity must be considered. However, for the bioimaging applications, inorganic nanoparticles are the preferred nanocarrier type, as carbon nanotubes and quantum dots exhibit more serious neurotoxic effects.

## 3. Nanomedicine in Central Nervous System Disorders

**Figure 3:**
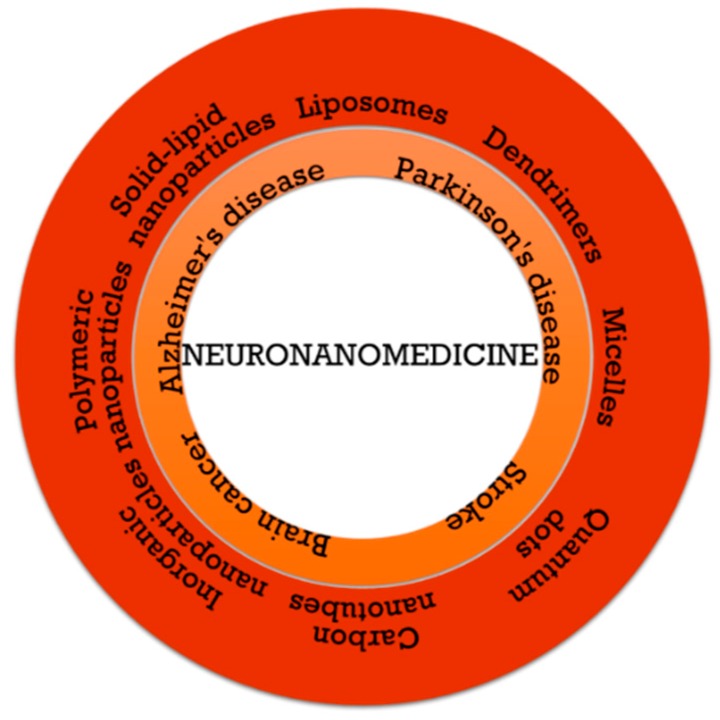
The applications of nanomaterials as nanocarriers in brain cancer, neurodegenerative diseases, and stroke therapy.

The central nervous system comprises hundreds of various highly organized subtypes of neurons and glia, thus being the most complex and specialized body system. Consequently, diseases associated with the central nervous system are equally complex, causing various diagnostically definitive disruptions in behavior [76]. As nanomaterials are considerably advantageous in regard to their effective targeting, non-invasiveness, stability, biodegradability, and possibility to control the encapsulation and release of the drugs, they have gained a great interest in the area of neuromedicine [48]. Therefore, significant advances have been made in the development of nanotherapeutics capable of crossing the BBB for the diagnosis and/or treatment of the central nervous system disorders (Figure 3) [77], which will be thoroughly described (Table 2 and Table 3).

### 3.1. Brain Cancer

The transport of anti-cancer drugs through the BBB for the treatment of brain tumors remains one of the major challenges in brain cancer therapy. Thus, the development of nanotechnology-based strategies for an efficient brain uptake and controlled release of the active compounds is essential. The characteristics of the nanocarriers mostly depend on the cancer type, tumor characteristics, stage, and location [78].

Novel approaches for the diagnosis of brain cancer involve the use of nanoparticles, liposomes, micelles, and quantum dots for various neuroimaging techniques. Therefore, silica shells double-coated with semiconducting polymer layers were synthesized for fluorescence and photoacoustic brightness imaging [79]. Moreover, phosphonate polyethylene glycol and cyclo RGD functionalized iron-oxide nanoparticles [80] or bovine serum albumin and tumor-specific folic acid [81] functionalized iron-oxide nanoparticles were applied for magnetic resonance imaging. Another strategy for the diagnosis of glioblastoma involves coating gold nanoparticles with the CBP4 peptide for an enhanced binding to the CD133 biomarker [82]. Furthermore, liposomes incorporating heptamethine cyanine dye IR780 [46] and iron-oxide nanoparticles and a near-infrared fluorescence dye [83] were studied for near-infrared fluorescence imaging and magnetic resonance imaging. Magnetic resonance imaging using gadolinium-incorporated micelles was used for the quantitative hemorrhage-risk evaluation due to the correlation between the extravasation of micelles and the hemorrhagic edema site [84]. Another study focused on the use of polyethylene glycol-coated quantum dots as neuroimaging systems at the tumor site through IVIS imaging system. However, to acquire the images, it was necessary to remove the skulls of the mice [85].

Studies regarding the treatment of brain cancer focused on the delivery of various anti-cancer drugs using nanocarriers. Specifically, poly(lactide-*co*-glycolic) acid nanoparticles containing doxorubicin, cisplatin, and boldine resulted in an efficient internalization into the glioma cells, inducing cytotoxic effects [86] and an effective target-specific delivery [87]. Other polymeric nanocarriers for the treatment of brain cancer include polyethylene glycol and poly(ω-pentadecalactone-*co*-*p*-dioxanone) [88] or polyethylene glycol and poly(lactic-*co*-glycolic) acid [89] block copolymer nanoparticles which led to an improved drug release efficiency and a decrease in tumor size. Moreover, the administration of amphiphilic polymer-lipid nanoparticles containing docetaxel led to the in vivo accumulation at the tumor site, with an enhanced tumor growth inhibition and increased median survival compared to the equivalent clinical dose of docetaxel solution [90]. Liposomal formulations have also been utilized for the delivery of various anti-cancer drugs, namely methotrexate [91], doxorubicin, erlotinib [92], 5-fluorouracil [93], and paclitaxel [94]. The passage through the BBB has been enhanced by coating the liposomes with different molecules. The results showed an extended blood-circulation time by coating with polyethylene glycol [91], an enhanced translocation across the BBB by attaching transferrin for receptor targeting [92,93], and a higher accumulation of the nanocarriers at the tumor site by conjugating the glucose-vitamin C complex [94]. As previously mentioned, the conjugation of dendrimers with molecules such as polyethylene glycol and glioma homing peptides [95], or sialic acid, glucosamine, and concanavalin A could significantly increase tumor penetration and consequently the amount of drug at the tumor site and reduce the efflux of the nanocarriers [96]. Other chemically functionalized nanocarrier systems for targeting brain tumors are micellar formulations for the delivery of curcumin [97] and multi-walled carbon nanotubes, which showed an increased tumor uptake for the targeted systems [98]. Additionally, the in vitro cytotoxic effects of USPIONs on glioblastoma multiforme was evaluated using rat CNS-1 cell cultures. Results showed that the USPIONs entered the cells through clatherin-coated pits which were further internalized in vacuoles and the effects of USPIONs on cell viability and mitopotential were dose- and time-dependent [99].

### 3.2. Neurodegenerative Diseases

Neurodegenerative diseases are increasingly prevalent age-dependent disorders which represent a major threat to human health. The most common neurodegenerative diseases are Alzheimer’s disease, Parkinson’s disease, Huntington’s disease, and multiple sclerosis, each characterized by their own pathophysiology, from memory and cognitive impairments to motor dysfunctions, affecting the ability to move, speak, or breathe. As effective treatment strategies are urgently needed, extensive studies regarding the potential of nanotechnology have been performed [100].

Characterized by conformational changes of native proteins which lead to the aggregation and formation of insoluble amyloid fibrils, neurodegenerative diseases therapy mostly relies on the development of adequate platforms that could detect the amyloid formations [101]. Neuroimaging applications of nanotechnology for diagnosing these diseases mainly focus on the use of inorganic nanomaterials as imaging nanocarriers, including iron-oxide nanoparticles [102], gadolinium-based nanoparticles [103], and plasmonic nanoparticles [101]. Furthermore, the administration of carbon nanotubes conjugated with the Pittsburgh Compound B could lead to a more effective early diagnosis of Alzheimer’s disease and therapy monitoring [104].

The treatment of neurodegenerative diseases through nanotechnology approaches focuses on both organic and inorganic nanocarriers. Polyethylene glycol and/or poly(lactic-*co*-glycolic) acid biodegradable polymeric nanoparticles functionalized with specific antibodies [105,106] or oligopeptide drugs [107] have been applied for the elimination of amyloid fibrils in Alzheimer’s disease. Furthermore, in vitro studies showed that the use of polymeric nanocarriers for the delivery of curcumin resulted in an enhanced drug delivery with reduced oxidative stress, inflammation, and plaque load [108]. In vivo studies regarding the administration of chitosan nanoparticles for the delivery of saxagliptin demonstrated the capacity to prevent premature release and enhanced site targeting compared to the equivalent dose of free saxagliptin in solution [109]. The treatment of Parkinson’s disease through the administration of polymeric nanocarriers has also been studied. Thus, administering chitosan nanoparticles through the intranasal route for the delivery of selegiline [110] and pramipexole [111] increases the brain targeting efficiency and the amount of drug reaching the brain by decreasing the pre-systemic metabolism. Moreover, the in vitro and in vivo studies on the delivery of apolipoprotein E and α-mangostin by using phosphatidic acid-conjugated liposomes [112] and transferrin-modified liposomes [113], respectively, confirmed the potential for an enhanced BBB permeation and efficient drug delivery. Polyamidoamine dendrimers containing carbamazepine [114] and micelles containing curcumin [115] have been reported for the treatment of Alzheimer’s disease. Inorganic nanomaterials, such as gold nanoparticles and carbon nanotubes have been studied for reducing the β-amyloid induced Alzheimer’s disease. Therefore, the functionalization of gold nanoparticles with β-amyloid specific peptides led to an enhanced BBB permeation for the in vitro models [116] and adsorption of berberine onto the surface of the multi-walled carbon nanotubes increased the amount of the drug in the brain [117]. Additionally, gold nanoparticles in the form of l-DOPA functionalized multi-branched nanoflower-like gold nanoparticles have shown potential in the treatment of Parkinson’s disease [118]. Furthermore, improvement of motor dysfunctions and decreased apoptosis could be achieved through the administration of cerium oxide nanoparticles, which have the capacity to protect neurons against reactive oxygen species-induced damage [119].

### 3.3. Stroke

As it can cause disability or even death, stroke represents a major concerning medical emergency. There are two types of stroke characterized by different mechanisms for triggering. Thus, a cerebral blood vessel blockage is the main cause for the ischemic stroke, while the hemorrhagic stroke is triggered by the rupture of the cerebral blood vessel [120]. Although the prevalence of the ischemic stroke is higher, causing inflammation, damages to the neurovascular unit and even neurological death, the available treatments are limited [120,121]. Current strategies for both emergency treatment and recovery focus on the development of inorganic and organic nanoparticles, such as metal and metal oxide nanoparticles, polymeric nanoparticles, and liposomes [122,123].

One pilot study performed on a gel brain phantom, New Zealand rabbits, and a middle-aged human male reported the potential of administering SPIONs for the rapid diagnosis of the emergent stroke through microwave imaging. Injection of the nanoparticles resulted in the possibility to approximate an area of reduced attenuation difference associated with ischemic hypo-perfusion of the left carotid circulation [124].

Studies for stroke therapy reported the use of poly(lactic-*co*-glycolic) acid nanoparticles functionalized with chlorotoxin as a targeting ligand for the co-delivery of Lexiscan and Nogo-66, for the simultaneous improvement of the BBB permeability and effective targeting of the stroke site. This system has proved its potential for stroke therapy as results showed an increased stroke survival [125]. Furthermore, polyethylene glycol conjugated polyamidoamine dendrimers have been applied as nanoplatforms for the delivery of drugs that could eliminate blood clots from the vessel [126]. The delivery of siRNA and endothelial progenitor cells represent a promising strategy for ischemic stroke therapy. Therefore, SPIONs have been used as nano-vehicles for gene therapy and for cell tracking, simultaneously. In addition, to further increase the migration and the survival rate of the cells, hif-prolyl hydroxylase 2 silencing might be used [127].

### 3.4. Clinical Applications

Due to the unmet medical need in the treatment of brain diseases, AstraZeneca has been focusing on key aspects of neurodegenerative diseases, analgesia, and psychiatry. Recent works have been studying the MEDI1814, a monoclonal antibody as a potential disease-modifying treatment for Alzheimer’s disease. This strategy is based on the ability of MEDI1814 to selectively target β-amyloid 42 [128], which is highly associated with Alzheimer’s disease [129]. As early trials have proved that MEDI1814 can reduce the levels of the β-amyloid 42, AstraZeneca and Lily are co-developing it as part of the BACE alliance.

Scientists at BiOasis have developed the xB^3^ patented platform, formerly known as Transcend-peptide, for applications in neuromedicine. This platform involves the use of a human transport protein found circulating at low levels in the blood, which has shown high efficiency in delivering molecules across the BBB through receptor-mediated transcytosis. Preclinical studies proved the capacity of xB^3^ to transport molecules such as monoclonal antibodies, enzymes, small-interfering RNA, and other types of gene therapies into the brain, thus having a great potential to treat brain cancers and metabolic and neurodegenerative diseases.

Moreover, the Cerense^®^ technology (Pharmidex Pharmaceutical Service, London, UK), formerly LipoBridge^®^ by Genzyme, and G-Technology^®^ (to-BBB, Leiden, The Netherlands), using BBB targeting delivery systems are in the clinical development pipeline. On one hand, the Cerense^®^ technology utilizes short-chain oligoglycerophospholipids that can transiently open the BBB tight junctions and facilitate the drug transport. On the other hand, the G-Technology comprises liposomes coated with polyethylene glycol and covered with glutathione to facilitate drug transport across the BBB through receptor-mediated transcytosis [130].

## 4. Challenges and Limitations

The continuous emergence of nanotechnology in the biomedical field has raised some concerns regarding the potential health risks as opposed to the associated benefits. In certain conditions, the numerous advantageous physicochemical properties of nanomaterials, such as reduced size, reactive surface, high surface to volume ratio, or tunable shape might cause serious toxic effects [131].

On one hand, their unique tunable characteristics might provoke unpredictable biological responses when introduced into the body [131]. As it leads to highly reactive and colloidal instability, the high surface to volume ratio is a cause for nanomaterial aggregation [132]. Consequently, as they form clusters no longer in the nanoscale, the cellular uptake is decreased, and apoptosis might be induced. Furthermore, research studies suggest the capacity of nanomaterials to translocate from the administration site to secondary vital organs, including the brain, liver, heart, lungs, and kidneys [133]. As nanomaterials could exert serious toxic effects at the accumulation site, thorough in vivo experiments that investigate organ toxicity and carcinogenicity are vital [134].

Moreover, the administration of nanomaterials for neurological purposes might lead to immediate and direct neurotoxic effects. Therefore, oxidative stress, induced cell apoptosis and necrosis, and immune responses and inflammation as main neurotoxic effects, result in the activation of specific signaling pathways that will further affect the function of the BBB. Additionally, the neurotoxic effect could directly alter the structure and activity of the neurons, or, due to the activation of glial cells and interactions between glial and neuronal cells, it might result in a cascade of effects. Neurotoxic effects manifest immediately or after certain periods of time, leading to reversible or permanent consequences that can affect parts of the nervous system or the whole system [135]. Table 4 summarizes the main neurotoxic effects of the previously described nanocarriers.

Another challenge for the administration of nanomaterials in biological systems is the formation of the biocorona on the surface. The biocorona might lead to alterations of the physicochemical properties, functionality, and biodistribution, and induce highly toxic effects [136].

On the other hand, the application of nanotechnology in the field of biomedicine is limited by the lack of standardized model systems, experimental assays, and in vivo monitoring systems to accurately determine the toxic effects of nanomaterials. Current BBB models involve the use of primary co-cultures of mouse brain endothelial cells and astrocytes, primary mono-, co-, and triple-cultures of rat endothelial cells/astrocytes/pericytes, bovine co-cultures of endothelial cells and astrocytes, porcine monocultures of endothelial cells, and human cultures using either the cCMEC/D3 endothelial cell line or stem cells. As the BBB is a highly dynamic barrier and its properties change in various physiological and pathological conditions, these models should be further refined to allow for the translation of results to the in vivo settings [137]. Additionally, the mechanisms underlying the impact of nanomaterials on biological systems are incompletely understood and further research work is fundamental in order to limit the risks associated with the neuronanomedicinal strategies [131]. One possible solution to accelerate the transition from the in vitro and animal model studies is the implementation of recently developed techniques of lab-on-a-chip and organ cultures. This strategy could allow for more rapid and accurate results regarding the efficiency of the nanomedical approach and the safety of applying it to the human body.

## 5. Conclusions and Perspectives

Neuronanomedicine has merged the fields of neuroscience and nanotechnology, through which nanomaterials, nanoformulations, and nanofacturing processes are effectively applied in neurology to further understand the physiological and pathological mechanisms. By this means, novel strategies for diagnosing and treating the disorders of the central nervous system have emerged. Specifically, nanotechnology mostly focuses on the development of organic and inorganic nanocarriers, but also on the use of biological entities, such as viral vectors or extracellular vesicles, that could efficiently deliver imaging and therapeutic agents across the BBB, into the brain parenchyma. Although studies are showing promising results, there are several limitations regarding the immediate and long-term interactions of these nanocarriers with the biological tissues. Additional coatings using extracellular matrix-derived polymers or anti-microbial materials and antibiotic treatments might offer possible solutions to reduce neurotoxic effects and to avoid bacteria adherence on the surface of the nanocarrier.

Besides the development of standardized experimental assays, future perspectives might also focus on the development of nanotechnology approaches for neuronal cell regeneration and reconstruction. Furthermore, there are promising possibilities in the field neurosurgery that could benefit from the advantages of nanotechnologies. One example is represented by the implementation of nanorobotics in neurosurgery that involves several manipulation technologies, such as the assembly of nanosized objects and biological cell and molecules manipulation. Moreover, this could also represent a solution for the early diagnosis and therapy monitoring of central nervous system disorders.

## Figures and Tables

**Table 1:** The main advantages, disadvantages, and surface functionalization strategies for the organic and inorganic nanocarriers.

Nanocarrier Type	Advantages	Disadvantages	Surface Functionalization Strategies
Polymeric nanoparticles	biocompatibility, biodegradability, drug protection, ease of preparation, good tolerance controlled pharmacokinetics tunable physicochemical properties	neurotoxicity	polysorbate 80 RVG29 peptide anti-Aβ1-42 antibody monoclonal antibody (OX26) anti-Aβ (DE2B4) g7 ligand TGN peptides QSH peptides l-valine chlorotoxin
Solid-lipid nanoparticles	biocompatibility, high physical stability, bioavailability, drug protection, strict control of release, ease of preparation, good tolerance, and biodegradability without generating toxic by-products no neurotoxic effects reported hydrophobic drug entrapment efficiency lipophilicity possibility of passively cross the BBB	reduced hydrophilic drug entrapment efficiency sterilization difficulties	apolipoprotein E
Liposomes	possibility of entrapping both hydrophilic and hydrophobic compounds improved drug protection and targeting efficiency lipophilicity possibility of passively cross the BBB	neurotoxicity physicochemical instability tendency of fusion rapid clearance sterilization difficulties	phosphatidylserine-targeting antibody polyethylene glycol transferrin PFVYLI peptide penetratin peptide glucose-vitamin C complex phosphatidic acid apolipoprotein E
Dendrimers	possibility of entrapping both hydrophilic and hydrophobic compounds biodegradability stimuli-responsiveness enhanced targeting efficiency	neurotoxicity synthesis variability rapid clearance organ accumulation	polyethylene glycol glioma homing peptides sialic acid glucosamine concanavalin A
Micelles	no neurotoxic effects reported improved drug bioavailability physicochemical stability sustained and controlled release	use only for lipophilic drugs low drug loading capacity	Tween 80
Inorganic nanoparticles	unique optical, electrical, and magnetic properties tunable size, shape, composition, structure, and porosity prolonged enhanced permeability and retention effect enhanced on-demand drug release by applying external stimuli (near-infrared radiation and magnetic field)	neurotoxicity high tendency of aggregation non-degradableorgan accumulation need further functionalization for BBB crossing	cyclo RGD peptides phosphonate polyethylene glycol bovine serum albumin folic acid CBP4 peptide KLVFF and LPFFD peptides CLPFFD peptides l-DOPA hif-prolyl hydroxylase 2 silencing
Carbon nanotubes	unique structure, exceptional electrical, mechanical, optical, and thermal properties, and high surface area	neurotoxicity need further functionalization for BBB crossing	Pittsburgh Compound B polysorbate and phospholipid coating
Quantum dots	exceptional optical and electrical properties	neurotoxicity need further functionalization for BBB crossing	polyethylene glycol asparagine-glycine-arginine peptides

**Table 2:** A summary of the nanotechnology-based neuroimaging approaches for the diagnosis of brain cancer, neurodegenerative diseases, and stroke.

Central Nervous System Disorder	Nanocarrier Type	Functionalization	Imaging Agent	Neuroimaging Technique	Study Model	Reference
Brain cancer	silica shells double coated with semiconducting polymer layers	cyclo RGD peptides	-	fluorescence and photoacoustic brightness imaging	in vitro—4T1 human breast cancer epithelial cells in vivo—tumor-bearing female mice	[79]
	iron oxide nanoparticles	phosphonate polyethylene glycol and cyclo RGD peptides	-	magnetic resonance imaging	in vitro—U87-MG cells in vivo—tumor-bearing nude mice	[80]
		bovine serum albumin and tumor-specific folic acid	fluorescein isothiocyanate	magnetic resonance imaging	in vitro—U251 cells	[81]
	gold nanoparticles	CBP4 peptide	fluorescein isothiocyanate	confocal microscopy	in vitro—U373 human glioma cells	[82]
	liposomes	-	heptamethine cyanine dye IR780	near-infrared fluorescence imaging	in vitro—U87MG human glioma cells and T98G human glioblastoma cells in vivo—glioblastoma mouse models	[46]
		phosphatidylserine-targeting antibody	iron oxide nanoparticles and a near-infrared fluorescence dye	near-infrared fluorescence imaging and magnetic resonance imaging	in vitro—U87MG human glioma cells in vivo—tumor-bearing nude mice	[83]
	micelles	-	gadolinium	magnetic resonance imaging	in vivo—Wistar male rats	[84]
	quantum dots	polyethylene glycol and asparagine–glycine–arginine peptides	-	IVIS imaging	in vitro—primary rat BCECs, astrocytes and C6 glioma cells in vivo—Sprague–Dawley male rats	[85]
Neurodegenerative diseases	gadolinium-based nanoparticles	KLVFF and LPFFD peptides	-	fluorescence microscopy	in vivo—APPswe/PS1A246E/TTR mouse model	[103]
	carbon nanotubes	Pittsburgh Compound B	gadolinium complexes	single photon emission computed tomography/computed tomography and γ-scintigraphy	in vivo—female C57BL/6 mice	[104]
Stroke	Iron-oxide nanoparticles	-	-	microwave imaging	in vitro—gel brain phantom in vivo—New Zealand rabbits and a middle-aged human male	[124]

**Table 3:** A summary of the nanotechnology-based treatment strategies for brain cancer, neurodegenerative diseases, and stroke.

Central Nervous System Disorder	Nanocarrier Type	Functionalization	Active Compound	Study Model	Reference
Brain cancer	poly(lactide-*co*-glycolic) nanoparticles	poloxamer 188	doxorubicin	in vitro—U-87 MG, ATCC cell line	[86]
		-	cisplatin and boldine	in vivo – tumor-bearing swiss albino mice	[87]
	polyethylene glycol and poly(ω-pentadecalactone-*co*-*p*-dioxanone) nanoparticles	-	VE822	in vitro—RG2 cells in vivo —Tumor-bearing male Fischer 344 rats	[88]
	polyethylene glycol and poly(lactic-*co*-glycolic) acid nanoparticles	RVG29 peptide	docetaxel	in vitro—C6 cells in vivo—tumor-bearing adult Sprague–Dawley male rats	[89]
	amphiphilic polymer-lipid nanoparticles	polysorbate 80	docetaxel	in vitro—MDA-MB-231 cells in vivo—tumor-bearing severe combined immune deficiency mice	[90]
	liposomes	polyethylene glycol	methotrexate	in vivo – male Sprague–Dawley rats	[91]
		transferrin and PFVYLI peptide	doxorubicin and erlotinib	in vitro—U87 tumor cells, brain endothelial cells, and glial cells	[92]
		transferrin and penetratin peptide	5-fluorouracil	in vitro—U87 tumor cells and brain endothelial cells	[93]
		glucose-vitamin C complex	paclitaxel	in vitro—C6 cells in vivo—C6 glioma-bearing Kunming mice	[94]
	dendrimers	polyethylene glycol and glioma homing peptides	-	in vitro—U87MG cells in vivo—U87MG tumor-bearing BALB/c nude mice	[95]
		sialic acid, glucosamine, and concanavalin A	paclitaxel	in vitro—U373MG human astrocytoma cell line in vivo—Sprague–Dawley rats	[96]
	micelles	Tween 80	curcumin	in vitro—G422 cells	[97]
	multi-walled carbon nanotubes	Angiopep-2	-	in vitro—primary porcine brain endothelial cells and primary rat astrocytes in vivo—GL261 glioma-bearing female C57/Bl6 mice	[98]
	USPIONS	-	-	in vitro—rat CNS-1 cells	[99]
Neurodegenerative diseases	polyethylene glycol nanoparticles	anti-Aβ1-42 antibody	-	in vivo—NIHS adult male mice	[105]
	poly(lactic-*co*-glycolic) acid nanoparticles	monoclonal antibody (OX26) and anti-Aβ (DE2B4)	-	in vitro—porcine brain capillary endothelial cells	[106]
	poly(lactic-*co*-glycolic) acid nanoparticles	g7 ligand	curcumin	in vitro—primary hippocampal cultures from rat brains	[108]
	polyethylene glycol-polylactic acid nanoparticles	TGN peptides and QSH peptides	coumarin-6 and H102	in vitro—brain endothelial cells in vivo—5XFAD transgenic mice	[107]
	chitosan nanoparticles	L-valine	saxagliptin	in vivo—female Wistar rats	[109]
		-	selegiline	ex vivo—male Sprague–Dawley rats	[110]
		-	pramipexole dihydrochloride	ex vivo—goat nasal mucosa in vivo—male Sprague–Dawley rats	[111]
	liposomes	phosphatidic acid and apolipoprotein E	quercetin and rosmarinic acid	in vitro—brain microvascular endothelial cells and Aβ1-42-insulted SK-N-MC cells	[112]
		transferrin	α-mangostin	in vitro—brain endothelial cells in vivo—Sprague–Dawley rats	[113]
	polyamidoamine dendrimers	-	carbamazepine	ex vivo—human red blood cells in vitro—N2a cell linein vivo—zebrafish	[114]
	micelles	-	curcumin	in vitro—U87MG cell line in vivo—female Sprague–Dawley rats	[115]
	gold nanoparticles	CLPFFD peptides, neutral methoxy terminated polyethylene glycol ligands, and negatively-charged monosulfonated triphenylphosphine ligands	-	in vitro—porcine brain capillary endothelial	[116]
		L-DOPA	-	in vitro—human brain endothelial cell line hCMEC/D3, brain microvascular endothelial cells, and mouse microglia N9 cell line	[118]
	multi-walled carbon nanotubes	polysorbate and phospholipid coating	berberine	in vitro—human red blood cells and SH-SY5Y cells in vivo—male Wistar rats	[117]
	cerium oxide nanoparticles	-	-	in vivo—adult male Wistar rats	[119]
Stroke	poly(lactic-*co*-glycolic) acid nanoparticles	chlorotoxin	Lexiscan and Nogo-66	in vivo—male C57BL/6 mice	[125]
	polyamidoamine dendrimers	polyethylene glycol	-	in vitro—rat primary astrocytes and mouse brain endothelial cells in vivo—male C57BL/6 mice	[126]
	iron oxide nanoparticles	hif-prolyl hydroxylase 2 silencing	siRNA	in vivo—female BALB/c nude mice	[127]

**Table 4:** A summary of the main neurotoxic effects of the organic and inorganic nanocarriers for BBB crossing [135].

Nanocarrier Type	Neurotoxic Effect
Polymeric nanoparticles	neuronal apoptosis; neuroinflammation; increased oxidative stress
Liposomes	necrosis; neuroinflammation; hemorrhage; macrophage infiltration
Dendrimers	cell proliferation and migration inhibition; abnormal mitochondrial activity; apoptosis; affected neuronal differentiation; increased oxidative stress; DNA damage; decreased locomotor function
Gold nanoparticles	increased oxidative stress; cognition defects; astrogliosis
Silver nanoparticles	increased oxidative stress; apoptosis; necrosis; neuroinflammation
Iron oxide nanoparticles	synaptic transmission and nerve conduction alterations; neuroinflammation; apoptosis; macrophage infiltration
Titanium oxide nanoparticles	increased oxidative stress; neuroinflammation; apoptosis; synaptic transmission alterations and plasticity; genotoxicity
Silica nanoparticles	cognitive dysfunctions and impairment; neurodegeneration; synaptic transmission alterations
Carbon nanotubes	neuroinflammation; cell proliferation inhibition; apoptosis; increased oxidative stress; mitochondrial membrane potential reduction; lipid peroxidization; astrocyte function reduction; neurobehavioral toxicity
Quantum dots	increased oxidative stress; cell function damage; neurobehavioral toxicity; cognitive impairment
